# Process Evaluation of a Sport-Based Supportive Care Intervention for Testicular Cancer Survivors: A Mixed Methods Study

**DOI:** 10.3390/cancers14112800

**Published:** 2022-06-04

**Authors:** Anika R. Petrella, Catherine M. Sabiston, Roxy H. O’Rourke, Daniel Santa Mina, Robert J. Hamilton, Andrew G. Matthew

**Affiliations:** 1 Cancer Division, University College London Hospitals NHS Foundation Trust, London NW1 2PG, UK; anika.perella1@nhs.net; 2 Faculty of Kinesiology and Physical Education, University of Toronto, Toronto, ON M5S 2W6, Canada; roxy.orourke@mail.utoronto.ca (R.H.O.); daniel.santamina@utoronto.ca (D.S.M.); 3 The Princess Margaret Cancer Centre, University Health Network, Toronto, ON M5G 2C1, Canada; rob.hamilton@uhn.ca (R.J.H.); andrew.matthew@uhn.ca (A.G.M.)

**Keywords:** testicular cancer, survivorship, physical activity, sport, feasibility and acceptability, mixed methods

## Abstract

**Simple Summary:**

Testicular cancer is the most common cancer diagnosed in adolescent and young adult men. The disease, fortunately, has a high survival rate, meaning that many survivors need long-term, follow-up care. A lack of engagement in such care, however, continues to be a problem for this population. One promising model of supportive care that appears acceptable and appealing to young men is the community-based model. Yet much of the community support research is observational and descriptive. In this study, a five-week community-based sports health promotion intervention named, *The Ball’s in Your Court* was developed and piloted. Findings suggest that it can be used to engage young men in supportive care and may be effective at improving health and wellness throughout survivorship in this population.

**Abstract:**

Testicular cancer survivors report unmet supportive care needs that are associated with poorer physical and mental health, yet engagement in traditional supportive care is low. *The Ball’s in Your Court* intervention was designed to engage testicular cancer survivors in supportive care by leveraging a community-based sport and exercise model. Age-appropriate, gender-sensitized, and disease specific elements were reflected in the intervention design, setting, content, and delivery. The intervention included five weekly health promotion sessions among a group of testicular cancer survivors. The purpose of this study was to explore the intervention’s (i) feasibility and acceptability, (ii) effects on testicular cancer survivors’ perceived health, and (iii) gain feedback for intervention refinement. A total of 10 testicular cancer survivors participated in the pilot and completed questionnaires on demographics, cancer history, perceived health, and physical activity behavior at baseline (pre-intervention) and perceived health and satisfaction with intervention components (post-intervention). Open-ended feedback surveys were collected after each weekly session and researcher field notes were recorded by three members of the study team. One month following the intervention, a focus group was conducted with intervention participants. All participants were satisfied with the intervention. Content analysis of the qualitative data supported intervention acceptability. Visual analysis conducted at the individual level indicated that perceived health either remained stable or improved from pre- to post-intervention. *The Ball’s in Your Court* intervention provides a feasible and acceptable approach for the delivery of supportive care aimed at improving testicular cancer survivors’ health and wellness. Recommendations for intervention refinement were provided and require future examination.

## 1. Introduction

There is a growing recognition that young people with cancer require tailored medical approaches and supportive care following diagnosis and treatment [1,2,3]. Supportive care refers to any provisions offered to cancer survivors with the objective of addressing quality of life challenges, which may include preventing or treating cancer-related side effects or addressing psychosocial concerns [4]. Although supportive care has become more widely available in major cancer centres, current programming may be inadequate in meeting the needs of young people diagnosed during adolescence and young adulthood (i.e., between the ages of 15 to 39) [5,6,7]. This concern has led to the development of dedicated survivorship care programs that are tailored to young adult survivors; however, engagement from young men, particularly testicular cancer survivors, has been low [8]. Poor uptake in supportive care research and usual care by this population does not appear to be due to a lack of need. Testicular cancer survivors are a growing population (5-year survival rate of over 95%) who have reported unmet supportive care needs following diagnosis and curative treatment that relate to physical and mental health and wellness [9,10,11,12,13].

Young adult cancer survivorship research seldomly focuses on the experiences of men; however, three studies exploring supportive care needs of testicular cancer survivors have been reported. In 2004, Jonker-Pool and colleagues [14] focused on testicular cancer survivors’ sexual health needs and reported that these needs were unmet in 67% of their sample. Bender and colleagues [9] reported that 65% of the 204 testicular cancer survivors surveyed had at least one unmet need, mainly concerning body image, stress, identity following cancer, fear of recurrence, and/or financial support. Even when survivors reported their needs as met, they expressed strong interest in engaging in services if made available, suggesting a desire for additional support. Preferred features of such support included ways to manage side effects and connecting with other survivors, whereas information on available support groups and online chat rooms was not as well endorsed [9]. These findings highlight the desire for informational and social support that are delivered in an appealing way for testicular cancer survivors. 

Another study by Smith and colleagues [12] reported that among 244 testicular cancer survivors, unmet needs ranged from no unmet needs to as many as 34 unmet needs, with an average of nearly five unmet needs (e.g., stress reduction, connectedness with other survivors, sexual health, fear of cancer recurrence, disclosure). Importantly, most survivors reported one or more unmet supportive care need(s), which were specific to existential survivorship issues (e.g., fear of cancer recurrence, life stress). A higher total number of unmet needs was associated with psychological distress, poorer health-related quality of life, depressive symptoms, poorer social functioning, and worse mental health [12]. These findings indicate a need for additional supportive care programming to address a wide range of physical and psychosocial survivorship challenges. However, the optimal way of providing supportive care to this population is still unknown. 

In an effort to engage men in health promotion interventions, sport-based intervention models have been successfully developed and delivered in middle aged men [15,16,17,18], young men [19] and prostate cancer survivors [20,21,22]. These novel interventions engage men in multiple group-based intervention components targeting physical health (e.g., sport play, sideline drills, diet and nutritional support) and mental health (e.g., facilitated social support, psychoeducation on stress reduction and mental health). These interventions have been shown to be feasible, acceptable, and effective in improving the physical and mental health of men [16,18,19,20]. Furthermore, a group-based sport program delivered within a community setting was appealing to participants [18,23]. However, it is unknown whether young men with cancer would find a community sport-based intervention appealing. 

To address this gap in knowledge, Petrella and colleagues [24,25] explored the association between physical activity and survivorship experiences of young men diagnosed and treated for testicular cancer. First, a positive association between psychological needs and self-rated physical and mental health was observed, with exercise meditating that relationship [24]. Second, testicular cancer survivors’ attitudes towards, and preferences for, sport-based supportive care were explored [25]. A sample of testicular cancer survivors considered a sport-based supportive care model to be an appropriate approach to supportive care that could potentially break down existing barriers to participation and provide an avenue for regaining control of their health after cancer [25]. Survivors indicated a preference for tailored supportive care that was offered outside of the hospital setting, in the evening, and that included strength training and embedded psychoeducation. These preferences directly informed the design and development of a tailored supportive care intervention named *The Ball’s in Your Court* (see Table 1 for intervention overview). 

*The Ball’s in Your Court* intervention was inclusive of three core components (resistance training, sport play, psychoeducation via a workbook) that were designed to be age-appropriate, gender-sensitized, and disease-specific. The resistance training component was entrusted to an expert strength and conditioning specialist who delivered a standard protocol, and the sport play to a varsity coach who led the drills and games. The psychoeducational workbook was developed based on the available literature within young adult cancer survivorship and findings from interviews with testicular cancer survivors [25], as well as the primary researchers’ expertise working with this clinical population as a psychotherapist. During the development phase, the content of each individual workbook module (e.g., managing difficult emotions) was reviewed by a clinical psychologist who was a member of the study team. Once these three components were established, *The Ball’s in Your Court* intervention was pilot tested. 

Implementing a community program is important for knowledge mobilization in cancer and physical activity research [26], and more work is needed to explore the impact of community-based physical activity (i.e., sport and exercise) on patient-reported outcomes, such as perceived physical and mental health. Interventions are complex by nature and consist of multiple interacting components with the intention to achieve a desired outcome [27]. Drawing on the Medical Research Council (MCR) framework for intervention development and evaluation, examining the feasibility and acceptability of these types of interventions is needed [28]. The feasibility of conducting a multi-component intervention refers to the whether or not the research procedures and intervention components can be carried out as intended [29]. Acceptability refers to “the extent to which people delivering or receiving a healthcare intervention consider it to be appropriate, based on anticipated or experienced cognitive and emotional responses to the intervention” [30]. Feasibility and acceptability can be assessed quantitatively (e.g., attendance, dropout, measuring satisfaction) and qualitatively by probing individuals’ appraisals of the intervention [28]. Given the complexity of supportive care interventions, it is essential that individual components are evaluated for optimization, as guided by the Multiphase Optimization Strategy (MOST).

### The Present Study

The aim of the current study was to (i) gather insights into the feasibility and acceptability of *The Ball’s in Your Court* intervention reflected by concurrent and retrospective perceptions of the intervention and its exercise, sport, and supportive care components; (ii) examine changes in testicular cancer survivors’ perceived physical and mental health from pre- to post-intervention; and (iii) gather recommendations for intervention refinement.

## 2. Materials and Methods

*The Ball’s in Your Court* intervention was pilot-tested at a university campus in an urban Canadian setting. The intervention ran for five weeks and consisted of weekly 90-min group-based sessions (resistance training and sport play) supplemented by a tailored supportive care workbook. Feasibility was assessed in terms of attendance, drop out, and the challenges related to implementing the pilot, and acceptability was explored in terms of satisfaction and the appropriateness of the intervention. Changes in perceived physical and mental health at the individual level were examined using a single-subject design, which is consistent with patient-centered care [31] and allows for the evaluation of subtle changes within individuals over time [32,33,34]. Recommendations for intervention refinement were explored qualitatively. 

### 2.1. Study Design 

The current study included data collection pre-intervention, weekly following the completion of each session, post-intervention, and at one month follow-up. A baseline survey was collected pre-intervention to gain demographic and cancer-specific information, as well as to obtain perceptions of physical and mental health. Weekly open-ended feedback survey responses were collected at the end of each session, and researcher field notes (e.g., visual observations and informal feedback from participating survivors) were documented throughout the intervention delivery phase. Attendance and reasons for missing sessions were recorded weekly. Following completion of the intervention, a post-intervention survey was collected that measured program satisfaction and perceived physical and mental health. Finally, focus group data were collected at one-month follow-up. Data were analyzed separately and then integrated in the results section to provide a detailed representation of feasibility, acceptability, and potential effect of the pilot intervention, as well as feedback on intervention components and recommendations for future delivery [35]. The study protocol was granted Research Ethics Board approval prior to study initiation. 

### 2.2. Participants

A sample size of 12 men was the maximum number of participants for this pilot intervention to ensure adequate and fair training and sport play time. Using convenience sampling methods, testicular cancer survivors interested in participating in *The Ball’s in Your Court* intervention responded to study advertisements that were posted on social media or distributed through local community survivorship and wellness programs. Survivors were given additional study information and were screened for eligibility by a member of the study team. Survivors were eligible if they met the following inclusion criteria: (i) male aged 18 years or older; (ii) had a known diagnosis of testicular cancer (not limited by stage, time since diagnosis, or treatments received); (iii) had completed treatment a minimum of eight weeks ago; (iv) had no contraindications to exercise (e.g., responded “no” to all questions on the Physical Activity Readiness Questionnaire (PAR-Q+)) [36]; (v) received medical clearance; and (vi) were proficient in English. Men who met the criteria and provided informed consent were enrolled in the study. 

The intervention involved attending 2-h sessions weekly for five weeks. Pre-intervention baseline questionnaires were emailed to survivors one week prior to initiating the intervention. Participants were instructed to complete within-session evaluations immediately following each session and completed a post-intervention questionnaire. After the completion of the intervention, participants were invited to take part in an audio-recorded focus group at one-month follow-up. Researcher field notes were also taken at each session and during the focus group.

### 2.3. Measures

#### 2.3.1. Pre- and Post-Intervention Survey

Participants completed a self-report questionnaire at baseline that included demographic variables (age, ethnicity, relationship status, employment status, and education) and relevant cancer-related variables (date of diagnosis, tumor type, stage, and treatments received). Additional descriptive measures included baseline exercise behavior, which was assessed using the Godin Leisure Time Exercise Questionnaire (GLTEQ) [37,38]. Survivors were classified as meeting current exercise guidelines (e.g., >150 min of moderate-to-vigorous exercise per week) [39]. 

Aligned with previous work [25,26], self-rated physical and mental health were assessed pre- and post-intervention using single-item questions (“In general, would you say your physical/mental health is; poor, fair, good, very good, excellent”). Responses were recorded on a 5-point Likert-type scale ranging from 1 to 5 [40,41,42]. Satisfaction was measured post-intervention using five items, whereby survivors indicated how satisfied they were with: the exercise component (gym), the sport component, psychoeducation component, and the full intervention, on a 5-point Likert scale ranging from 0 (very dissatisfied) to 4 (very satisfied). Feedback specific to needs satisfaction (e.g., “To what extent did this intervention meet your survivorship needs”) was also queried, with survivors answering either 0 (none of my needs were met), 1 (some of my needs were met), or 2 (all of my needs were met). 

#### 2.3.2. Weekly Open-Ended Survey

To evaluate feasibility and acceptability of the intervention components without recall bias, weekly surveys were administered to survivors immediately following the completion of each session. Participants were asked to provide feedback on the session through four open-ended questions: (i) Exercise Session (gym): What did you like and not like about today’s gym session; (ii) Sport: What did you like and not like about today’s basketball session; (iii) Psychoeducation Component: What did you like and not like about this week’s workbook chapter; (iv) Other: Please feel free to add any additional comments or suggestions related to the study or your experience participating in the study. 

#### 2.3.3. Focus Group

At one-month follow-up, survivors were invited to participate in a focus group discussion held locally to the intervention location. A semi-structured focus group guide with open-ended exploratory questions acted as a flexible script to direct the focus group. Questions explored survivors’ experiences, what they liked about the intervention, and how the intervention could be improved. Each of the intervention components (e.g., exercise, sport, and psychoeducation) were consistently probed individually. Perceived benefits of participating in the intervention were explored (e.g., “How have you benefited from participating in this study”), as well as whether those benefits were maintained. Finally, enablers and barriers to participation and adherence were queried (e.g., “Why did you initially want to participate” and “Describe any enablers and barriers to participation/adherence”). The focus group was conducted by one member of the research team (AP) who is a female with seven years of experience as a psychotherapist working with men with cancer, and who has previous experience in qualitative data collection. The focus group discussion was audio-recorded and transcribed verbatim, and all identifiable information was removed from the transcripts to ensure confidentiality. 

#### 2.3.4. Field Notes on Intervention Implementation

Field notes were recorded and collected weekly by three members of the research team involved in the implementation of the intervention [43]. Field notes included information on the delivery of the intervention, as well as participant behavior and informal feedback throughout each session. The research team also kept track of attendance and queried participants’ reasons if absent. Moreover, ways in which the intervention could be modified or improved in the future were recorded. Field notes were used to supplement all other data by providing additional context regarding the participating survivors and their experiences, and to support critical reflection on the part of the researchers [44]. 

### 2.4. Data Analysis

Descriptive statistics (means, standard deviations, or frequencies) for participant characteristics and satisfaction scores were calculated using R (version 3.5.3) [45] summary tools [46]. Visual analysis using ggplot2 [47] explored within-subject changes in self-rated physical and mental health from pre- to post-intervention. Visual analysis, which is a foundational investigation of single-subject data, was conducted following established guidelines [48,49]. The mean for each physical and mental health (y-axis) was graphed against time (pre-intervention/post-intervention) (x-axis), and connected by a line. Change in scores from pre- to post-intervention, and the direction of change, were visually inspected, and change scores were calculated for participants who showed change over time. 

Deductive content analysis was used to analyze qualitative data [50,51]. Data were analyzed separately, following Elo and Kyngäs’s [51] three-phased approach to content analysis (preparation, organization, and reporting), beginning with the weekly open-ended survey responses. The field notes were analyzed second to continue with the temporal assessment (concurrent reflections), and the focus group transcript was analyzed last, demonstrating a retrospective reflection on acceptability [30]. During the preparation phase, A.R.P. read through the data (i.e., open-ended responses, field notes, and focus group transcript) several times. A categorization matrix was then developed based on the research questions and all three data types were coded using the same matrix. During the organizing phase, data were reviewed and coded according to categories defined by the categorization matrix and only those aspects of the data that fitted the matrix were included [51]. The minimal data that were discordant with the categorization matrix were not reported in the results of the current study. A second independent coder (R.H.O.) repeated the process of reading and organizing the data according to the categorization matrix. All categories and representative data were discussed by A.R.P., R.H.O., and C.M.S., and consensus on the final conclusions was reached through discussion and by revisiting the transcript where needed [50,51]. 

Quantitative results and qualitative findings were then integrated and interpreted using a weaving approach to develop a richer, more complex analysis of the feasibility and acceptability of the intervention, as well as individual level changes in perceived health and recommendations for intervention refinement [35]. 

## 3. Results

Recruitment spanned six weeks to achieve the recruitment target of 12 testicular cancer survivors. Of the twelve survivors who expressed interest, one interested survivor was unable to attend due to scheduling conflicts and a second became unreachable after initial contact. The remaining ten testicular cancer survivors were screened, met the eligibility criteria, and provided consent. The 10 survivors who participated in the intervention are identified using numbers one through ten (e.g., Participant 1, … Participant 10), and are described using four-year age ranges to ensure anonymity (see Table 2 for demographic and baseline characteristics). The age of participants ranged from 24 to 42 years (Mage = 32.7, SD = 6.41 years). Participants were predominantly Caucasian (80%), college or university educated (90%), and did not have any previous basketball experience barring school play.

No participants dropped out of the study and no adverse events were reported as a result of participation. Attendance varied week-to-week (50% to 100%), and one participant, who was absent in the final week, did not complete the post-intervention survey. All absences were attributed by participants to either work conflicts or non-cancer-related illness (i.e., cold or flu). Although all participants were invited to participate in the post-intervention focus group, only six of the ten participants were able to attend due to scheduling conflicts. 

Satisfaction with *The Ball’s in Your Court* intervention was generally high. Two thirds of participants reported being very satisfied and the remaining third somewhat satisfied with the intervention overall. All 9 participants who completed the post-intervention survey reported being satisfied with the exercise and sport components, with 56% and 44% reporting that they were very or somewhat satisfied for each component. Satisfaction with the workbook component varied, with 45% satisfied and just over 44% expressing ambivalence (i.e., neither expressing satisfaction nor dissatisfaction). All participants reported having their survivorship needs met as a result of engaging in the intervention, with 44% and 56% reporting that some or all of their needs were met.

To differentiate between the qualitative data sources reported below, acronyms are used to indicate data drawn from weekly open-ended surveys (e.g., WS1…WS5) and the focus group (FG). Context provided by research field notes are indicated in the text.

### 3.1. The Integrated Intervention 

“Just feels really great getting to know guys that have been through it all, we are all having a great time I think. Overall this is fantastic”. (WS3).

Participants appraised the *The Ball’s in Your Court* intervention as acceptable overall. Participants attributed their willingness to participate to their recognition of the value of a tailored survivorship intervention. During the focus group discussion, one survivor stated that, “these activities cater to the mental and physical state of a survivor” (FG) and that they should be integrated early on in survivorship. Participants also wrote that the “membership to the gym was great” (WS2) and, as indicated in the field notes, many of the men told the primary researcher that they used the facilities outside of the session time, primarily on weekends. Based on researcher field notes, one survivor informed the group that he had the confidence to join a gym closer to his house prior to completing the intervention and another survivor reported losing 10 pounds by the one-month follow-up. 

Participants approved many intervention delivery characteristics, such as frequency (once a week), duration (two hours), and location (university athletics center). However, the start time of 5 p.m. was identified as a potential barrier to engagement and participants suggested starting at 6pm or later. During the focus group, participants discussed a more flexible, drop-in design to accommodate varying work schedules; however, concerns around losing connectedness were raised and this idea was dropped by participants in favor of maintaining a structured intervention. Specific to intervention length, participants communicated a desire for a longer intervention and focus group discussions generated the recommendation of at least six to eight weeks for future interventions. 

The opportunity to connect with other testicular cancer survivors was identified as an enabler to program participation. When asked during the focus group discussion if the intervention could be inclusive of male participants of other cancer types (e.g., leukemia, lymphoma, thyroid, etc.), one survivor answered, “It is not the same... it is different drugs, different regimes” (FG) and another added that when he had talked to men with other cancers, “It is like you are talking to each other but it is like two different wave lengths” (FG). Participants explained that testicular cancer was a connecting factor, writing that there was “a quiet understanding” (WS1) among each other. Participants added that regardless of any differences in each other’s cancer journey (e.g., treatments, disease severity), there was a shared bound, “we all understand where we are coming from”. (FG). It was then agreed that it would be their preference to keep the intervention focused on testicular cancer. In addition, participants wanted social time and activities dedicated to getting to know each other more quickly, with socializing embedded throughout the intervention (e.g., icebreaker exercises). Specifically, participants requested organized social events after the session to provide additional opportunities to talk to one another. Nonetheless, this intervention appeared to quell some of the feelings of isolation and to be of value to supportive care “...having this (program) embedded in your follow-up (care) would be amazing” (FG) in order to help reduce those feelings of isolation because “… meeting other testicular cancer survivors makes it less isolating” (WS3).

Overall, the intervention was perceived positively, as one survivor stated, “it gave me a boost going back into work and you know physically, get in that physical groove again. I think it kind of perks you up mentally as well” (FG). Another survivor agreed, stating, “I have more energy for sure” (FG) and “...when you start to get in better shape you just feel better about yourself. Definitely more control” (FG). During the focus group a survivor explained “...I am more capable than what I think... I thought the first day I was going to be asking for oxygen” (FG). Immediately, another survivor cut in and said, “You know how that Scotiabank (commercial) says, you are richer than you think. You are stronger than you think man” (FG). Researcher field notes commented on the supportive atmosphere throughout intervention implementation and focus group discussions, and participants noted “everyone was very welcoming and friendly” (WS2) and that there was “good support from everyone” (WS5). The intervention was noted as being “great for comradery and team building” (WS4) and field notes indicated the use of humor to discuss their experiences, with one participant writing “a lot of guys connect well by simply cracking jokes” (WS5). 

### 3.2. The Exercise Component 

“It gave me confidence in my body again. I guess now I can raise the bar in my expectations of what I can do”. (FG).

The strength and conditioning component of the intervention was generally positively appraised. Participants reported enjoying the individually tailored nature, as stated by one participant: “I liked the exercises. Everything was modifiable for different levels/experiences. I already see a sense of progression in my physical capacity” (WS3). The opportunity to work with the strength and conditioning coach was valued by participants, as one participant stated that in the past “I did not have any program to follow, I would have liked to have one (prior to surgery)” (FG). Another survivor wrote that the exercise component “made me realize there were some physical motions that I thought I could not do BUT, I could” (WS1). The juxtaposition of individualized programs while exercising as a group was also viewed favorably, with one survivor stating “group exercise is much more enjoyable than alone” (WS2). 

Constructive feedback included a desire for more “…education on training” (FG), “more time to explore the other machines” (WS4), and “…more variety in terms of the workout” (FG). Researchers noted observing progression from week to week in participants’ comfort with the exercises and noted improvements in technique; however, participants wrote during the last session that “the exercises have become a little repetitive, but they were useful and challenging” (WS5). Research field notes indicated that participants were quiet and focused during the strength and conditioning component. Space within the gym was a noted issue by researchers (field notes) and participants, “did not like the lack of space and equipment but we were able to manage” (WS3). Some sessions felt rushed, with one survivor requesting “more time to complete all the workout” (WS3). Although none of the participants who participated in the focus group felt that the exercise intervention was too hard, one survivor thought “...the intensity might scare people away” (FG), adding that “even though it was a ton of fun, (we) might want to scale it down a little bit” (FG). Feedback via the survey highlighted this concern, with one survivor reporting, “heavy weight and less rest time was really challenging” (WS4) and another survivor articulated, “I didn’t like how out of shape I am” (WS4) in reference to his reflection on the intensity of the sessions. 

### 3.3. The Sport Component 

“I liked every aspect of it. Felt amazing to just play a team sport again, especially with fellow survivors” (WS2).

Although satisfaction with the sport component matched satisfaction with the exercise component, qualitative data emphasized participants’ preference for playing sport above all else. The sport component (i.e., basketball drills and game) was viewed as being most engaging and fun, as well as the main draw for participation. As one survivor stated, “it is just fun to like play sports. Like be a little competitive and run around” (FG). Sports aided in “...building rapport with each other” (FG) and when asked about the inclusion of varsity athletes, participants reported that their presence enhanced the overall experience. One survivor stated, “I thought University students would be weird or take it as a joke. They just wanted to play. That is what I liked about it” (FG) and another wrote that it was “great to have the varsity team push us and lighten the mood. Encouragement helped” (WS2). In addition, participants touched on liking being “pushed to work hard and not treated differently just because we went through cancer” (WS2). Although basketball was well received, with participants writing that it was “interactive & engaging” (WS2), a concern that other participants may “...not join the program... like oh I do not play basketball” (FG) was raised. Focusing exclusively on basketball was discussed as a potential barrier to participation and one survivor suggested, “a way around that may be if you mixed up the sports a little bit” (FG), by suggesting that a multi-sport program may “...attract a broader group of people” (FG). Multiple participants suggested incorporating “different sports and activities” (WS1) and that soccer specifically “might be a more universal sport” (WS2) to focus on. Participants also suggested that there be an “alumni group” (FG) that could act as peer mentors to new participants, as well as transition the group into a community sports league as “… a testicular cancer survivorship sport team… that connects the guys so it is more grass roots” (FG). 

Overall, researcher field notes commented on the high energy and fun atmosphere during the sport component. Observations documented in researchers’ field notes mentioned varsity players providing tailored instruction to participants, remarking on individual improvements from week to week, and engaging with participants in high fives and positive verbal support that was encouraging to each other. 

### 3.4. The Workbook: An Ambivalent Component of the Intervention 

Views on the workbook component of the intervention were mixed. Researchers’ field notes indicated that participants often reported having not completed the assigned chapter due to a lack of time or motivation to do so. One survivor articulated, “I would really enjoy making more time at the end of the session for the psychoeducation modules” (WS2). Participants touched on time since diagnosis and treatment as a factor related to the relevance of the workbook. One survivor stated, “being 3-years post-treatment I don’t identify as easily with a lot of the content” (WS5) while others stated that the content was “reflective of my situation” (WS3), “I liked that it really represents what I experienced” (WS3). Participants indicated that they would have liked to receive this workbook at the beginning of their cancer journey as the workbook was “good at identifying some strategies to manage emotions” (WS5). However, one survivor “found it a bit difficult to figure out how to use some of the resources at the back of the book” (WS5). This is suggestive of a need for more facilitation from the psychotherapist and was reflected in the field notes. Specific to the delivery format, participants unanimously agreed that a hard copy was better than an electronic copy and that the current 5.5 × 8.5 inch size of the workbook was ideal. 

Participants identified missing content and made suggestions for future iterations of the workbook. The content was perceived as being surgery- and chemotherapy-focused, and was therefore missing information specific to radiation therapy. Another survivor indicated that he was looking for “…some input on the effects of the steroids on mood” (WS3) and another stated “having an understanding of the long term, what things I should be looking for would be super helpful” (FG). Another survivor added, “...anything in the book that can highlight stuff that you are like oh I never even thought about that” (FG) would be helpful throughout survivorship. A thorough discussion was had around the inclusion of “...basic testicular cancer statistics” (FG), but participants disagreed, with one stating “statistics are not always going to apply to you” (FG), and adding that they may be anxiety provoking for some men. Information seeking specific to survivorship needs varied among participants, with some men reporting a desire for more information and others not wanting to engage with the available information. As one survivor underlined, “I am not really seeking (information) unless something becomes a problem” (FG). This finding speaks to the tailored and individual support needs of these men.

### 3.5. Single-Subject Analysis of Self-Rated Health 

Self-rated physical and mental health scores at pre-and post-intervention are presented in Table 3. Visual analysis was completed and graphs are available from the first author. Inspection of self-rated physical health indicated that Participant 2 improved from pre- to post-intervention (Δ 1.00), while all other participants remained stable. Inspection of self-rated mental health indicated that Participant 4 (Δ 1.00), as well as Participant 7 (Δ 1.00) improved over time, while all other participants remained stable. Of note, the mean score for self-rated physical health was higher than that for self-rated mental health at baseline and post-intervention. 

## 4. Discussion

The current study explored the feasibility and acceptability of a sport-based supportive care intervention for testicular cancer survivors, as well as individual level changes in the perceived health of participants. Recommendations for intervention refinement and optimization were also gained from the ten testicular cancer survivors who participated in the five-week pilot intervention. Findings from this study provide support for the feasibility and acceptability of a group, sport- and exercise-based supportive care model that engages young men after diagnosis, and also provides insights into the potential benefit of this model on survivors’ perceived health. 

### 4.1. Feasibility and Acceptability 

Research procedures (recruitment, data collection, attendance, drop out) and intervention components (exercise, sport play, and psychoeducation via workbook) were generally feasible. Challenges associated with recruiting young adult cancer survivors [52] and young men more generally [53] have been acknowledged. The current study successfully recruited 10 testicular cancer survivors over a six-week period. Attendance varied week-to-week, although 60% of participants attended 75% or more of the sessions and missed sessions were due to illness or work conflicts. These findings may be due to the time of year that this pilot intervention was run (fall) and the life stage of the participants (e.g., early career). No participants dropped out of the study; however, one participant did not return the post-intervention questionnaire. Time constraints were an issue, with the allotted time for each component appearing to be insufficient to complete in full. Future designs should consider reducing structured content. However, for the workbook component, a lack of structured time to discuss the content and potential lack of homework fidelity likely compromised this component’s effectiveness. Overall, the pilot was successfully executed; however, issues relating to feasibility were present and should be considered when interpreting the findings and in any planning for future research.

Acceptability of an intervention and its individual components has implications for successful implementation and overall effectiveness. If an intervention is considered by the target population to be acceptable, they are more likely to adhere to the intended design and are thus more likely to receive the anticipated benefit [30]. The acceptability of a supportive care intervention for testicular cancer survivors is important given the identified demographic and disease-related characteristics (e.g., age, life-stage, masculinity, self-reliance, physical limitations) that may impact on survivors’ need for, and engagement in, supportive care. Upon integration of the findings from this study, a multidimensional acceptability model was identified. Specifically, Sekhon et al. [30] suggest that there are seven component constructs that capture the key dimensions of acceptability. These dimensions include affective attitudes (i.e., how a participant feels about the intervention), ethicality (i.e., goodness-of-fit with an individual’s value system), burden (i.e., the perceived amount of effort required to participate), opportunity costs (i.e., loss of benefit, profit, or value due to participation), intervention coherence (i.e., understanding of the intervention and how it works), self-efficacy (i.e., an individual’s confidence in ability to perform behavior(s) required for participation), and perceived efficacy (i.e., the perceived likelihood that the intervention will achieve its aim). 

Effective attitudes towards the intervention overall and the individual components were generally positive, with survivors reporting that a sport-based intervention supported their physical and mental health, while providing an opportunity to meet other survivors. This is consistent with the notion that men connect by doing, supporting the benefit of engaging men in activities built into the intervention [54]. In newly formed groups of men, it may be particularly important to place an emphasis on the active components of an intervention rather than on social aspects, which may be off-putting for men [54]. Once the group is more established, a candid discussion about health and illness management can occur, which was articulated by participants in this study. Positive attitudes towards testicular cancer-specific intervention were expressed and these findings are consistent with the call for tailored supportive care for young people with cancer [55,56] and further support the acceptability of intervention components in the context of delivery. Attitudes relating to the benefits of physical and mental health and wellness as a result of group-based physical activity is consistent with group-based activity among women with cancer [57,58].

*The Ball’s in Your Court* intervention appeared to fit well with survivors’ existing value systems, speaking to the dimension of ethicality [30]. The exercise component was a direct request from testicular cancer survivors in previous work [25,59]; and is consistent with physical activity preferences among young men in the general population [19]. The value placed on building strength among these young men may be based in social norms where men have been shown to idealize a well-toned muscular physique [60,61,62]. The sport component also exhibited a good fit with the participating survivors’ values, likely due to sport being a historically male-dominated environment that fosters masculine characteristics and is one of the most common leisure time activities among young men [63,64]. The competitive element of the intervention appeared to resonate with survivors, which is a consistent element of acceptability among other sport-based, gender-sensitized pieces of research [15,18]. Finally, multiple survivors identified the workbook as being representative of their experience. 

Feedback on delivery characteristics highlighted the potential burdens or barriers associated with participation in this intervention that may impact on feasibility and acceptability. Survivors identified the intervention’s start time as a barrier to participation and also touched on the need to balance priorities and work schedules, yet their motivation for initial and continued participation appeared to outweigh these burdens. These findings are consistent with those reported among breast cancer survivors who participated in a group-based physical activity intervention within the community [65]. Specifically, breast cancer survivors reported barriers to engagement related to travel and managing competing priorities, and they reported being motivated by gaining social support, feeling a sense of fulfillment and acquiring health benefits [65]. Specific to intervention coherence and individuals’ self-efficacy, survivors expressed a desire for additional training during the exercise component and concerns were raised regarding low self-efficacy in basketball being a potential barrier. Furthermore, survivors reported having difficulties with some of the resources in the workbook, suggesting that additional facilitation is needed to increase acceptability. While understanding of the intervention and individual-level confidence around engaging in the behaviors required for participation was present, intervention refinement is needed to better support these dimensions of acceptability. 

The survivors who participated in *The Ball’s in Your Court* intervention perceived the intervention to be effective at addressing supportive care needs related to physical and psychosocial health and wellness. This is consistent with previous findings whereby men reported high levels of perceived effectiveness of intervention components aimed at supporting health and wellness [18,66]. In the current study, engaging testicular cancer survivors in sport may have helped to deconstruct contemporary views on supportive care among these young survivors and could demonstrate new contexts (e.g., outside of the hospital) in which these men can seek social support and supportive care. This may help to explain the high satisfaction scores found during retrospective assessment of the survivors’ experiences in the intervention. Satisfaction is a key construct in process evaluation and supports overall acceptability [27]. Consistent with previous research [15,18,66], men reported a higher satisfaction in the sport and exercise components, whereas satisfaction with the workbook component (i.e., psychoeducation) varied. Men also responded positively to the involvement of multiple professionals, which is notable feedback given the lack of engagement from men in healthcare [8] and the minimal reported reliance from testicular cancer survivors on health professionals [9]. Overall, findings are consistent with satisfaction scores reported in other sport-based health interventions for men [66] and the challenge is around the delivery of psychoeducation that is acceptable to a wide range of men. 

### 4.2. Changes in Self-Rated Health

Examination of individual level changes in perceived physical and mental health showed stability for the majority of survivors from pre- to post-intervention, whereas three participants showed an improvement. Numerous studies have provided evidence for the effectiveness of physical activity on improving physical and mental health and quality of life of cancer survivors [38,67,68]; however, few studies have examined the impact of sport [56,69]. Drawing from community health initiatives which have shown to be feasible, acceptable, and effective in improving men’s physical and mental health [16,18,19,66,70], it was hypothesized that a sport-based intervention would improve testicular cancer survivors’ perceived physical and mental health. Survivors who scored lower at baseline were among those who showed an improvement, supporting the notion that survivors with poorer perceived health have greater room to improve over time compared to those reporting higher functioning at baseline [71]. It is possible that a more sensitive measure of health and quality of life is needed to pick up subtle changes over time and that additional time points should be considered. 

The paucity of improvement in perceived physical health may be due, in part, to the length of the intervention. In order to see objective and perceived changes in physical fitness, a longer intervention should be tested [72]. Incorporating goal-setting into future interventions may also facilitate greater engagement in physical activity behavior and improved self-efficacy, as seen in other physical activity interventions for young adults [73]. The intervention setting (university athletics center) and the involvement of varsity players was reflected on positively by survivors; however, understanding its potential impact on survivors’ perceptions of physical health should be considered in future research. For example, survivors who perceive their cancer experience to be disruptive to educational or vocational goals may be at risk of engagement in a negative social comparison with the university athletes who have been unaffected by cancer and are functioning at peak performance. The improvements observed in perceived mental health suggest that intervention components may assist survivors in normalizing their diagnosis and in regaining a sense of control, which is consistent with the recommended aims of supportive care for young adults [4]. It is postulated that mental health would improve by engaging with other testicular cancer survivors [74]. Group dynamics researchers suggest that individuals with greater social bonds are more likely to adhere to a group physical activity intervention [75] and suggest that increasing cohesion may foster social connectedness [76]. Social connectedness could thus be supported by embedding team building strategies (e.g., creating a group name, providing group t-shirts, and engaging in shared goal setting) within program delivery to enhance group cohesion [77]. Additional research is needed to understand changes in perceived health, the timing of when they occur, and the links to specific intervention strategies.

### 4.3. Recommendations for Refinement

In recognizing the acceptability of tailored supportive care for testicular cancer survivors, a number of recommendations should be considered for future refinement and optimization. Firstly, to overcome the challenges related to attendance and therefore increase feasibility, interventions should be offered later in the evening; this recommendation is consistent with the physical activity intervention preferences reported among the cancer survivors [78]. Given the constraints on time and available resources, as well as the proof-of-concept design, this intervention ran for five weeks. Inconclusive evidence and a lack of recommended guidelines exist regarding intervention length required to promote improvement in relevant outcomes among cancer survivors. Of the limited research in testicular cancer survivorship, Adams and colleagues [79] reported improvements in testicular cancer survivors’ health-related quality of life following engagement in a 12-week exercise intervention. Furthermore, the Canadian Network for Mood and Anxiety Treatments guidelines [80] recommend that interventions targeting depression be no shorter than nine weeks in length. Thus, a longer intervention should be developed and tested. 

The current findings also highlight a need for additional opportunities for testicular cancer survivors to participate in the giving and receiving of social support. Sport is an environment where engaging in social support is socially acceptable [15,81]; however, additional opportunities outside of sport were requested by survivors. Embedding between-sessions and post-intervention peer support may enhance feelings of connectedness to, and support from, others and thus positively impact on health-related quality of life [78]. The development of a peer-support model was recommended by survivors and should be explored given the success in other clinical populations [82]. Survivors also requested more variety in both the exercise and sport components, which has been shown to positively influence exercise behavior [83,84]. Finally, given that the workbook was not consistently used by survivors, there is value in revisiting the delivery method and ensuring that survivors received adequate facilitation with this component. The Football Fans in Training intervention and its offspring have successfully included classroom discussions as a delivery method of supportive care and should be considered [15,85]. 

### 4.4. Limitations and Future Directions 

This study has several strengths. The intervention was evidence-based and was designed and developed in collaboration with testicular cancer survivors and community stakeholders [25,59] through a community-based participatory approach, which resulted in the first sport-based supportive care intervention tailored to the needs of this population. Unlike traditional supportive care interventions that focus solely on either physical activity [79] or psychosocial care [86], this intervention included physical and psychosocial components. The intervention was then implemented within the community setting (i.e., outside of the hospital), where it was designed to be offered, to observe its potential. This allowed for naturally occurring external variables to be present that may not have been in a hospital-based research lab setting [87]. This approach supports the potential transferable nature of the intervention to other universities and allows for practical refinement of the intervention to better meet the needs of this unique population. However, it should be noted that since this intervention was developed specifically for testicular cancer survivors, it may not be transferable to other groups of young male cancer survivors without adaptation. Lastly, exploring cost effectiveness was not within the scope of this project and therefore future research should aim to quantify the value of offering supportive care within a local university setting where ongoing access to resources may be available. 

While this study is the first to explore the feasibility, acceptability, and potential effectiveness of a sport-based supportive care intervention model for an understudied sample of cancer survivors (i.e., testicular cancer survivors), there are some limitations. The small sample of testicular cancer survivors were recruited using convenience sampling; therefore, self-selection bias naturally limits generalizability to the broader population of testicular cancer survivors. This is a common limitation of any single-subject design [49], and thus future research should aim to recruit a larger sample. This study also utilized self-report measures, which, although appropriate for assessing self-rated health, can be affected by recall and response bias (e.g., social desirability) and therefore additional measures of objective physical activity and clinical measures of physical and mental health could be considered.

Furthermore, multiple stakeholders were not integrated into the acceptability evaluation of the intervention, and the fidelity of the intervention was not examined. The short duration of the intervention (i.e., 5-weeks) may also have limited the potential for the intervention to have an effect on perceived health. A longer intervention design with additional data points and measures would be ideal for the evaluation of a future iteration of this intervention. Finally, the intervention setting was an urban center with extensive equipment and facilities. Future development, implementation, and process evaluation work should explore the feasibility, acceptability, and effectiveness of this model in different settings (e.g., rural) to understand potential intervention reach in supporting the needs of testicular cancer survivors across Canada. 

## 5. Conclusions

Taken together, results from this study provide initial support for the feasibility and acceptability of *The Ball’s in Your Court* intervention. Given that testicular cancer survivors are traditionally less likely to engage with supportive care services or rely on healthcare providers [9], the success of *The Ball’s in Your Court* intervention suggests that connecting survivors outside of the hospital in tailored, gender-sensitized programming will be feasible and well-received by testicular cancer survivors. These findings, along with previous research, highlight the role of sport as a feasible and acceptable starting point for future refinement and delivery of supportive care aimed at supporting long-term survivorship outcomes in men living with and beyond testicular cancer.

## Figures and Tables

**Table 1:** *The Ball’s in Your Court* intervention overview.

Intervention Design	Strength and Conditioning Component	Sport Component	Psychoeducational Workbook
Once per week 2-h session Lasting 5-weeks Provided gym membership for single academic term (4-months)	60-min Facilitated by strength and conditioning coach Consistent workout every week Workout included: ○ Warm-up (10-min): stretching, muscle activation, and movement preparation ○ Three circuits (15-min each): 2 to 3 exercises covering major muscle groups (4 sets × 10 repetitions) ○ Self-monitored weight and repetitions recorded in workbook ○ Individual exercises were modifiable	60-min Facilitated by varsity coach & three varsity players Basketball centered games (e.g., bump) and three-on-three basketball play Free to rest at any time	Tailored supportive care workbook Discussed during cool down and stretching Facilitated by a psychotherapist Four chapters: ○ Managing Side-Effects ○ Managing Difficult Emotions ○ Healthy Living ○ Defining the New ‘Normal’ One chapter each week assigned for homework Included: worksheets and links to additional resources Room for notes at the back of the workbook

**Table 2:** Demographics and baseline characteristics of participants (N = 10).

	Age within 4-Year Range	Relationship Status	Employment	Histology	Stage	Treatment(S) Received	Time Since Diagnosis (Years)	Meeting Exercise Guidelines	Attendance (%)
Participant 1	30–34	Living with life partner	Full-time	Non-seminomas	2	Surgery; chemotherapy	3.58	Yes	100%
Participant 2	35–39	Living with life partner	Full-time	Don’t know	2	Surgery; chemotherapy	6.33	No	80%
Participant 3	30–34	Single	Full-time	Seminomas	1	Surgery; radiation	8.00	Yes	100%
Participant 4	25–29	Married	Full-time	Non-seminomas	1	Surgery; chemotherapy	3.83	Yes	60%
Participant 5	40–44	Living with life partner	Part-time	Non-seminomas	1	Surgery	2.25	Yes	80%
Participant 6	35–39	Single	Disability leave	Non-seminomas	3 *	Surgery; Chemotherapy	1.00	Yes	100%
Participant 7	20–24	Living with life partner	Unemployed	Mixed germ cell	1	Surgery	0.25	Yes	50%
Participant 8	40–44	Living with life partner	Full-time	Seminomas	1	Surgery	24	Yes	75%
Participant 9	25–29	Living with life partner	Full-time	Non-seminomas	1	Surgery	0.25	Yes	50%
Participant 10	25–29	Married	Full-time	Seminomas	1	Surgery	0.50	No	50%

Note: Meeting exercise guidelines cutoff of ≥150 min of moderate to vigorous exercise per week. * Participant 6 self-reported as stage 4 (metastatic stage 3).

**Table 3:** Participants’ pre- and post-intervention self-rated physical and mental health scores (N = 10).

	Baseline Pre-Intervention		Post-Intervention	
	Self-Rated Physical Health	Self-Rated Mental Health	Self-Rated Physical Health	Self-Rated Mental Health
Participant 1	5	5	5	5
Participant 2	2	2	3	2
Participant 3	4	3	4	3
Participant 4	4	3	4	4
Participant 5	3	2	3	2
Participant 6	5	4	5	4
Participant 7	4	3	4	4
Participant 8	3	3	3	3
Participant 9	4	4	4	4
Participant 10	2	2	-	-
Mean (SD)	3.60 (1.07)	3.10 (0.99)	3.89 (0.78)	3.44 (1.01)

Note: Participant 10 did not complete the post-intervention survey.

## Data Availability

The data presented in this study are available on request from the corresponding author. The data are not publicly available due to the small sample size.

## References

[1] Barnett M., McDonnell G., DeRosa A., Schuler T., Philip E., Peterson L., Touza K., Jhanwar S., Atkinson T.M., Ford J.S. (2016). Psychosocial outcomes and interventions among cancer survivors diagnosed during adolescence and young adulthood (AYA): A systematic review. J. Cancer Surviv..

[2] Barr R.D., Ferrari A., Ries L., Whelan J., Bleyer W.A. (2016). Cancer in adolescents and young adults: A narrative review of the current status and a view of the future. JAMA Pediatr..

[3] D’Agostino N.M., Penney A., Zebrack B. (2011). Providing developmentally appropriate psychosocial care to adolescent and young adult cancer survivors. Cancer.

[4] National Cancer Institute Dictionary of Cancer Terms-Supportive Care. http://www.cancer.gov/publications/dictionaries/cancer-terms?cdrid=46609.

[5] Aubin S. (2011). Challenges conducting qualitative psychosocial research for adolescent and young adult patients and survivors. J. Adolesc. Young Adult Oncol..

[6] Sender L., Zabokrtsky K.B. (2015). Adolescent and young adult patients with cancer: A milieu of unique features. Nat. Rev. Clin. Oncol..

[7] Wilkins K.L., D’Agostino N., Penney A.M., Barr R.D., Nathan P.C. (2014). Supporting adolescents and young adults with cancer through transitions: Position statement from the Canadian Task Force on Adolescents and Young Adults with cancer. J. Pediatr. Hematol. Oncol..

[8] Kim P.Y., Loscalzo L.M.J. (2018). Gender in Psycho-Oncology.

[9] Bender J.L., Wiljer D., To M.J., Bedard P.L., Chung P., Jewett M.A.S., Matthew A., Moore M., Warde P., Gospodarowicz M. (2012). Testicular cancer survivors’ supportive care needs and use of online support: A cross-sectional survey. Support. Care Cancer.

[10] Fung C., Sesso H.D., Williams A., Kerns S.L., Monahan P., Abu Zaid M., Feldman D., Hamilton R.J., Vaughn D.J., Beard C.J. (2017). Multi-Institutional Assessment of Adverse Health Outcomes Among North American Testicular Cancer Survivors After Modern Cisplatin-Based Chemotherapy. J. Clin. Oncol..

[11] Schepisi G., De Padova S., De Lisi D., Casadei C., Meggiolaro E., Ruffilli F., Rosti G., Lolli C., Ravaglia G., Conteduca V. (2019). Psychosocial issues in long-term survivors of testicular cancer. Front. Endocrinol..

[12] Smith A.B., King M., Butow P., Luckett T., Grimison P., Toner G.C., Stockler M., Hovey E., Stubbs J., Hruby G. (2013). The prevalence and correlates of supportive care needs in testicular cancer survivors: A cross-sectional study. Psychooncology.

[13] Znaor A., Skakkebaek N.E., Rajpert-De Meyts E., Kuliš T., Laversanne M., Gurney J., Sarfati D., McGlynn K.A., Bray F. (2022). Global patterns in testicular cancer incidence and mortality in 2020. Int. J. Cancer.

[14] Jonker-Pool G., Hoekstra H.J., van Imhoff G.W., Sonneveld D., Sleijfer D.T., van Driel M.F., Koops H.S., van de Wiel H.B. (2003). Male sexuality after cancer treatment—needs for information and support: Testicular cancer compared to malignant lymphoma. Patient Educ. Couns..

[15] Gray C.M., Hunt K., Mutrie N., Anderson A.S., Leishman J., Dalgarno L., Wyke S. (2013). Football Fans in Training: The development and optimization of an intervention delivered through professional sports clubs to help men lose weight, become more active and adopt healthier eating habits. BMC Public Health.

[16] Gray C.M., Wyke S., Zhang R., Anderson A.S., Barry S., Brennan G., Briggs A., Boyer N., Bunn C., Donnachie C. (2018). Long-term weight loss following a randomised con-trolled trial of a weight management programme for men delivered through professional football clubs: The Football Fans in Training follow-up study. Public Health Res..

[17] Quested E., Kwasnicka D., Thøgersen-Ntoumani C., Gucciardi D., Kerr D.A., Hunt K., Robinson S., Morgan P.J., Newton R.U., Gray C. (2018). Protocol for a gender-sensitised weight loss and healthy living programme for overweight and obese men delivered in Australian football league settings (Aussie-FIT): A feasibility and pilot randomised controlled trial. BMJ Open.

[18] Seaton C.L., Bottorff J.L., Oliffe J.L., Jones-Bricker M., Caperchione C.M., Johnson S.T., Sharp P. (2017). Acceptability of the POWERPLAY Program: A Workplace Health Promotion Intervention for Men. Am. J. Men’s Health.

[19] Ashton L.M., Morgan P.J., Hutchesson M.J., Rollo M.E., Collins C.E. (2017). Feasibility and preliminary efficacy of the ‘HEYMAN’ healthy lifestyle program for young men: A pilot randomised controlled trial. Nutr. J..

[20] Bjerre E.D., Brasso K., Jørgensen A.B., Petersen T.H., Eriksen A.R., Tolver A., Christensen J.F., Poulsen M.H., Madsen S.S., Østergren P.B. (2018). Football Compared with Usual Care in Men with Prostate Cancer (FC Prostate Community Trial): A Pragmatic Multicentre Randomized Controlled Trial. Sports Med..

[21] Bruun D.M., Bjerre E., Krustrup P., Brasso K., Johansen C., Rørth M., Midtgaard J. (2014). Community-based recreational football: A novel ap-proach to promote physical activity and quality of life in prostate cancer survivors. Int. J. Environ. Res. Public Health.

[22] Uth J., Hornstrup T., Schmidt J.F., Christensen J.F., Frandsen C., Christensen K.B., Helge E.W., Brasso K., Rørth M., Midtgaard J. (2014). Football training improves lean body mass in men with prostate cancer undergoing androgen deprivation therapy. Scand. J. Med. Sci. Sports.

[23] Hunt K., Wyke S., Gray C.M., Anderson A.S., Brady A., Bunn C., Donnan P.T., Fenwick E., Grieve E., Leishman J. (2014). A gender-sensitised weight loss and healthy living pro-gramme for overweight and obese men delivered by Scottish Premier League football clubs (FFIT): A pragmatic randomised controlled trial. Lancet.

[24] Petrella A.R., Sabiston C.M., O’Rourke R.H., Santa Mina D., Matthew A.G. (2020). Exploring the survivorship experiences and prefer-ences for survivorship care following testicular cancer: A Mixed Methods Study. J. Psychosoc. Oncol. Res. Pract..

[25] Petrella A.R., Sabiston C.M., Vani M.F., Matthew A., Mina D.S. (2021). Psychological Needs Satisfaction, Self-Rated Health and the Mediating Role of Exercise Among Testicular Cancer Survivors. Am. J. Men’s Health.

[26] Phillips S.M., Alfano C.M., Perna F.M., Glasgow R.E. (2014). Accelerating translation of physical activity and cancer survivorship re-search into practice: Recommendations for a more integrated and collaborative approach. Cancer Epidemiol. Prev. Biomark..

[27] Moore G.F., Audrey S., Barker M., Bond L., Bonell C., Hardeman W., Moore L., O’Cathain A., Tinati T., Wight D. (2015). Process evaluation of complex interventions: Medical Research Council guidance. BMJ.

[28] Craig P., Dieppe P., Macintyre S., Michie S., Nazareth I., Petticrew M. (2008). Developing and evaluating complex interventions: The new Medical Research Council guidance. BMJ.

[29] Eldridge S.M., Lancaster G.A., Campbell M.J., Thabane L., Hopewell S., Coleman C.L., Bond C.M. (2016). Defining Feasibility and Pilot Studies in Preparation for Randomised Controlled Trials: Development of a Conceptual Framework. PLoS ONE.

[30] Sekhon M., Cartwright M., Francis J.J. (2017). Acceptability of healthcare interventions: An overview of reviews and development of a theoretical framework. BMC Health Serv. Res..

[31] Tate R.L., Perdices M. (2015). N-of-1 Trials in the Behavioral Sciences. The Essential Guide to N-of-1 Trials in Health.

[32] Naughton F., Johnston D. (2014). A starter kit for undertaking n-of-1 trials. Eur. Health Psychol..

[33] Hsia C.C., Mahon J.L., Seitelbach M., Chia J., Zou G., Chin-Yee I.H. (2016). Use of n-of-1 (single patient) trials to assess the effect of age of transfused blood on health-related quality of life in transfusion-dependent patients. Transfusion.

[34] McFadden T., Fortier M.S., Guérin E. (2017). Investigating the effects of Physical Activity Counselling on depressive symptoms and physical activity in female undergraduate students with depression: A multiple baseline single-subject design. Ment. Health Phys. Act..

[35] Fetters M.D., Curry L.A., Creswell J.W. (2013). Achieving Integration in Mixed Methods Designs-Principles and Practices. Health Serv. Res..

[36] Warburton D.E., Bredin S.S., Jamnik V.K., Gledhill N. (2011). Validation of the PAR-Q+ and ePARmed-X+. Health Fit. J. Can..

[37] Godin G. (2011). The Godin-Shephard leisure-time physical activity questionnaire. Health Fit. J. Can..

[38] Godin G., Shephard R.J. (1985). A simple method to assess exercise behavior in the community. Can. J. Appl. Sport Sci..

[39] Segal R., Zwaal C., Green E., Tomasone J., Loblaw A., Petrella T. (2017). The Exercise for People with Cancer Guideline Development Group Exercise for People with Cancer: A Clinical Practice Guideline. Curr. Oncol..

[40] Herman K.M., Hopman W.M., Sabiston C.M. (2015). Physical activity, screen time and self-rated health and mental health in Canadian adolescents. Prev. Med..

[41] Krause N.M., Jay G.M. (1994). What do global self-rated health items measure?. Med. Care.

[42] Picard M., Juster R.-P., Sabiston C.M. (2013). Is the whole greater than the sum of the parts? Self-rated health and transdisciplinarity. Health.

[43] Phillippi J., Lauderdale J. (2017). A Guide to Field Notes for Qualitative Research: Context and Conversation. Qual. Health Res..

[44] Braun V., Clarke V. (2013). Successful Qualitative Research: A Practical Guide for Beginners.

[45] Team R.R. A Language and Environment for Statistical Computing; R Foundation for Statistical Computing, Vienna, Austria. https://www.R-project.org/.

[46] Comtois D., Comtois M.D. (2016). Package ‘Summarytools’. https://mran.microsoft.com/snapshot/2016-12-03/web/packages/summarytools/summarytools.pdf.

[47] Wickham H. (2011). Ggplot2. Wiley Interdiscip. Rev. Comput. Stat..

[48] Lane J.D., Gast D.L. (2013). Visual analysis in single case experimental design studies: Brief review and guidelines. Neuropsychol. Rehabil..

[49] Barker J., McCarthy P., Jones M., Moran A. (2011). Single-Case Research Methods in Sport and Exercise Psychology.

[50] Elo S., Kääriäinen M., Kanste O., Pölkki T., Utriainen K., Kyngäs H. (2014). Qualitative content analysis: A focus on trustworthiness. SAGE Open.

[51] Elo S., Kyngäs H. (2008). The qualitative content analysis process. J. Adv. Nurs..

[52] Bradford N.K., Chan R.J. (2017). Health promotion and psychological interventions for adolescent and young adult cancer survivors: A systematic literature review. Cancer Treat. Rev..

[53] Ashton L.M., Morgan P.J., Hutchesson M.J., Rollo M.E., Young M.D., Collins C.E. (2015). A systematic review of SNAPO (Smoking; Nu-trition; Alcohol; Physical activity and Obesity) randomized controlled trials in young adult men. Prev. Med..

[54] Oliffe J.L., Rossnagel E., Bottorff J.L., Chambers S.K., Caperchione C., Rice S.M. (2020). Community-based men’s health promotion pro-grams: Eight lessons learnt and their caveats. Health Promot. Int..

[55] Fardell J.E., Patterson P., Wakefield C.E., Signorelli C., Cohn R., Anazodo A., Zebrack B., Sansom-Daly U. (2018). A Narrative Review of Models of Care for Adolescents and Young Adults with Cancer: Barriers and Recommendations. J. Adolesc. Young-Adult Oncol..

[56] Quinn G.P., Gonçalves V., Sehovic I., Bowman M.L., Reed D.R. (2015). Quality of life in adolescent and young adult cancer patients: A systematic review of the literature. Patient Relat. Outcome Meas..

[57] McDonough M.H., Sabiston C.M., Sedgwick W.A., Crocker P.R. (2010). Changes in Intrinsic Motivation and Physical Activity among Overweight Women in a 12-Week Dragon Boat Exercise Intervention Study. Women Sport Phys. Act. J..

[58] McDonough M.H., Sabiston C.M., Ullrich-French S. (2011). The Development of Social Relationships, Social Support, and Posttraumatic Growth in a Dragon Boating Team for Breast Cancer Survivors. J. Sport Exerc. Psychol..

[59] Petrella A.R. (2020). The Ball’s in Our Court: The Development of a Sport-Based Supportive Care Program for Testicular Cancer Survivors. Ph.D. Thesis.

[60] Sabiston C.M., Petrella A., Santa Mina D. (2019). Do General Perceptions of Social Support for Cancer Link to Supportive Strategies for Physical Activity among Men with Testicular Cancer?. J. Sport Exerc. Psychol..

[61] Bergeron D., Tylka T.L. (2007). Support for the uniqueness of body dissatisfaction from drive for muscularity among men. Body Image.

[62] Blashill A.J. (2011). Gender roles, eating pathology, and body dissatisfaction in men: A meta-analysis. Body Image.

[63] Blomberg C., Neuber N. (2016). Male identity; sport and health: Starting points for gender-sensitive support of boys and young men. Bundesgesundheitsblatt Gesundh. Gesundh..

[64] Riemer B.A., Visio M.E. (2003). Gender Typing of Sports: An Investigation of Metheny’s Classification. Res. Q. Exerc. Sport.

[65] Wurz A., St-Aubin A., Brunet J. (2015). Breast cancer survivors’ barriers and motives for participating in a group-based physical activity program offered in the community. Support. Care Cancer.

[66] Wyke S., Hunt K., Gray C.M., Fenwick E., Bunn C., Donnan P.T., Rauchhaus P., Mutrie N., Anderson A., Boyer N. (2015). Football Fans in Training (FFIT): A randomised controlled trial of a gender-sensitised weight loss and healthy living programme for men–end of study report. Public Health Res..

[67] Ferrer R.A., Huedo-Medina T.B., Johnson B.T., Ryan S., Pescatello L.S. (2010). Exercise Interventions for Cancer Survivors: A Meta-Analysis of Quality of Life Outcomes. Ann. Behav. Med..

[68] Sabiston C.M., Brunet J. (2011). Reviewing the Benefits of Physical Activity During Cancer Survivorship. Am. J. Lifestyle Med..

[69] Sabiston C.M., McDonough M.H., Crocker P.R. (2007). Psychosocial Experiences of Breast Cancer Survivors Involved in a Dragon Boat Program: Exploring Links to Positive Psychological Growth. J. Sport Exerc. Psychol..

[70] Johnson S.T., Stolp S., Seaton C., Sharp P., Caperchione C.M., Bottorff J.L., Oliffe J.L., Jones-Bricker M., Lamont S., Medhurst K. (2016). A men’s workplace health intervention: Results of the POWERPLAY program pilot study. J. Occup. Environ. Med..

[71] Hoyt M.A., Cano S.J., Saigal C.S., Stanton A.L. (2013). Health-related quality of life in young men with testicular cancer: Validation of the Cancer Assessment for Young Adults (CAYA). J. Cancer Surviv..

[72] Speck R.M., Courneya K.S., Mâsse L., Duval S., Schmitz K.H. (2010). An update of controlled physical activity trials in cancer survivors: A systematic review and meta-analysis. J. Cancer Surviv..

[73] Valle C.G., Tate D.F., Mayer D.K., Allicock M., Cai J. (2013). A randomized trial of a Facebook-based physical activity intervention for young adult cancer survivors. J. Cancer Surviv..

[74] Ryan R.M., Deci E.L. (2002). Overview of self-determination theory: An organismic dialectical perspective. Handb. Self-Determ. Res..

[75] Carron A.V., Shapcott K.M., Burke S.M. (2007). Group cohesion in sport and exercise: Past; present and future.

[76] Carron A.V., Spink K.S., Prapavessis H. (1997). Team building and cohesiveness in the sport and exercise setting: Use of indirect interventions. J. Appl. Sport Psychol..

[77] Eys M., Loughead T.M., Godfrey M. (2017). Group cohesion and athlete development. Routledge Handbook of Talent Identification and Development in Sport.

[78] Wong J.N., McAuley E., Trinh L. (2018). Physical activity programming and counseling preferences among cancer survivors: A sys-tematic review. Int. J. Behav. Nutr. Phys. Act..

[79] Adams S.C., DeLorey D.S., Davenport M.H., Fairey A.S., North S., Courneya K.S. (2018). Effects of high-intensity interval training on fatigue and quality of life in testicular cancer survivors. Br. J. Cancer.

[80] Ravindran A.V., Balneaves L.G., Faulkner G., Ortiz A., McIntosh D., Morehouse R.L., Ravindran L., Yatham L.N., Kennedy S.H., Lam R.W. (2016). Canadian Network for Mood and Anxiety Treatments (CANMAT) 2016 clinical guidelines for the management of adults with major depressive disorder: Section 5. Complementary and alternative medicine treatments. Can. J. Psychiatry.

[81] Eime R.M., Young J.A., Harvey J.T., Charity M.J., Payne W.R. (2013). A systematic review of the psychological and social benefits of participation in sport for children and adolescents: Informing development of a conceptual model of health through sport. Int. J. Behav. Nutr. Phys. Act..

[82] Sweet S.N., Noreau L., Leblond J., Martin Ginis K.A. (2016). Peer support need fulfillment among adults with spinal cord injury: Rela-tionships with participation; life satisfaction and individual characteristics. Disabil. Rehabil..

[83] Sylvester B.D., Curran T., Standage M., Sabiston C.M., Beauchamp M.R. (2018). Predicting exercise motivation and exercise behavior: A moderated mediation model testing the interaction between perceived exercise variety and basic psychological needs satis-faction. Psychol. Sport Exerc..

[84] Sylvester B.D., Standage M., McEwan D., Wolf S.A., Lubans D.R., Eather N., Kaulius M., Ruissen G.R., Crocker P.R.E., Zumbo B.D. (2015). Variety support and exercise adherence behavior: Experimental and mediating effects. J. Behav. Med..

[85] Van Nassau F., Van Der Ploeg H.P., Abrahamsen F., Andersen E., Anderson A.S., Bosmans J.E., Bunn C., Chalmers M., Clissmann C., Gill J.M.R. (2016). Study protocol of European Fans in Training (EuroFIT): A four-country randomised controlled trial of a lifestyle program for men delivered in elite football clubs. BMC Public Health.

[86] Heiniger L., Smith A.B., Olver I., Grimison P., Klein B., Wootten A., DPsych J.-A.M.A., Price M., McJannett M., Tran B. (2017). E-TC: Development and pilot testing of a web-based intervention to reduce anxiety and depression in survivors of testicular cancer. Eur. J. Cancer Care.

[87] Victora C.G., Habicht J.-P., Bryce J. (2004). Evidence-Based Public Health: Moving Beyond Randomized Trials. Am. J. Public Health.

